# Genetic variations and clinical significance in young-onset nasopharyngeal cancer: Analysis of EBV interaction with cellular receptor variants and viral glycoproteins

**DOI:** 10.1016/j.heliyon.2024.e41198

**Published:** 2024-12-13

**Authors:** Sulistyo Emantoko Dwi Putra, Farizky Martriano Humardani, Hikmawan Wahyu Sulistomo, Yulanda Antonius, Jonathan Jonathan, Riyan Charlie Milyantono, Artika Uthary, Risma Ikawaty

**Affiliations:** a Faculty of Biotechnology, University of Surabaya, Surabaya, 60292, Indonesia; b Magister in Biomedical Science Program, Faculty of Medicine Universitas Brawijaya, Malang, 65112, Indonesia; c Bioinformatics Research Center, Indonesia Bioinformatics and Biomolecular, Malang, 65162, Indonesia; d Faculty of Medicine, University of Surabaya, Surabaya, 60292, Indonesia

**Keywords:** EBV, Genetic variant, In-silico, Nasopharyngeal cancer, NRP1

## Abstract

Nasopharyngeal cancer (NPC), although rare in young individuals worldwide, is significantly influenced by the Epstein-Barr virus (EBV). Considering EBV's widespread prevalence, understanding its role in NPC's future occurrence, disease progression, clinical symptoms, metastatic tendencies, and prognosis is crucial. In this study, we extensively analyzed two young patients with NPC, who displayed distinct clinical features. We utilized Whole Exome Sequencing (WES), concentrating on EBV-interacting receptors, and applied advanced in silico methods for a deeper investigation. These methods included structural analysis via SWISS-MODEL, stability assessments using PremPS, and molecular docking studies with ClusPro. Our focus was to analyze genetic variants identified by WES and confirm EBV presence using RT-qPCR. Our comparative study between the two subjects showed that the first had milder symptoms and a lower metastasis than the second. In the first subject, we identified unique genetic variants: NRP1 c.536T > C (p.Val179Ala) and MYH9 c.4876A > G (p.Ile1626Val). Notably, the NRP1 p.Val179Ala variant caused structural changes leading to protein instability. Molecular docking suggested that this variant enhances interaction more than the wild-type. RT-qPCR validation of EBV showed lower levels in subject one (mutant-NRP1) compared to subject two (wild-type-NRP1). This finding implies that the p.Val179Ala variant in subject one could obstruct EBV entry, possibly leading to less severe clinical symptoms Our research provides new insights into the genetic factors influencing the clinical presentation of NPC, identifying promising targets for further research and therapeutic interventions. However, additional validation in a larger cohort is required to elucidate the broader impact of these genetic variants.

## Highlights

• The NRP1 allele G rs7079053 p.Val179Ala induces structural alterations and instability of NRP1 protein.
• The mutant NRP1 protein increases the binding energy, which may hinder the entry of EBV.
• The NRP1 allele G rs7079053 p.Val179Ala variant may show milder clinical manifestations and lower metastasis tendency.
• Qualitative and quantitative methods are promising for comparing proteins with unknown binding sites using blind docking.

## 1 Introduction

Nasopharyngeal cancer (NPC) is an epithelial tumor originating from the pharyngeal recess of the nasopharynx. Predominantly diagnosed around the age of 50, NPC is twice as prevalent in males as it is in females. The incidence rates among males display two distinct peaks: the first between 30 and 39 years of age, and the second between 50 and 59 years of age. Conversely, age-specific incidence rates are at their lowest in the younger population, specifically within the 10 to 29-year age range. Furthermore, epidemiological studies have identified individuals of Asian descent as being particularly vulnerable to NPC [[1], [2], [3]].

The Epstein-Barr virus (EBV) has a significant correlation with NPC. Lower EBV levels are associated with reduced progression and decreased survival rates, whereas higher EBV levels are linked to increased progression and improved survival rates [4]. This suggests that EBV plays a crucial role in the progression of NPC rather than in its initiation. Infection by EBV is managed by several receptors, including ITGB6, EphA2, NRP1, and MYH9 [5]. Despite this, there have been no clinical studies so far that have successfully determined the underlying mechanisms of these receptors in relation to EBV. The most recent research only identified a variant of ITGB6 present in NPC [6], yet the precise mechanism by which this gene variant contributes to the disease remains unknown.

In this study, we focused on two young patients diagnosed with NPC, who displayed distinct clinical manifestations and disease progression patterns despite having the same onset. To explore this, we employed whole exome sequencing (WES) to thoroughly investigate their genetic profiles, specifically targeting receptors interacting with EBV. Recognizing that genetic variants can significantly affect protein expression at multiple levels, possibly altering protein function to enhance, reduce, or completely eliminate its activity, we utilized an in-silico approach. This methodology was employed to assess the structural changes in the proteins and their interactions with EBV and receptors using molecular docking as influenced by the identified variants. To our knowledge, this is the first study in the context of NPC that integrates WES and in silico analysis. This novel approach aids in a better understanding of how genetic variations can influence protein function.

## 2 Materials and methods

### 2.1 Subject of study

This study involves subjects with young-onset NPC from East Java, Indonesia. The inclusion criteria specify that the patients must be diagnosed with young-onset NPC (under 20 years old), with confirmation of the diagnosis via CT-scan and Fine Needle Aspiration Biopsy (FNAB). The exclusion criteria encompass patients who have already undergone radiochemotherapy. Patient or guardian must give concern to including this study and this study was approved by Ibnu Sina General Hospital, Gresik, East Java, Indonesia (02/437.76/2023).

### 2.2 Sample preparation, analysis and validation of Epstein-Barr virus

A 5 mL blood sample was collected during a routine examination and was immediately frozen at −80 °C for preservation. Genomic DNA was then extracted from this sample using the FavorPrep™ Tissue Genomic DNA Extraction Mini Kit. In this study, we utilized specific primers for the validation of EBV, targeting the BamHI-W region and BALF5, as outlined in Supplementary Table 7. These primers and the methodology are based on previous research, referenced in studies [7,8], and the detection was performed using the Bio-Rad CFXDuet system. For sequencing, the DNA was processed using the Illumina NovaSeq 6000 system.

The WES data underwent comprehensive analysis. Sequence reads were aligned to the hg19 reference genome, followed by the removal of duplicate reads to enhance alignment precision. Variant calling was subsequently performed. To ensure the integrity and quality of the sequencing data, we employed MultiQC, a tool that integrates numerous bioinformatics quality control metrics from trimming through to variant calling [9]. The WES data thus obtained were thoroughly analyzed using MultiQC for comprehensive evaluation [9], reads were aligned to the hg19 reference genome, the alignments were refined by removing duplicate reads and variant calling using MultiQC [9]. Annotation was performed using Franklin (https://franklin.genoox.com/clinical-db/home), along with COSMIC, cBioPortal, Kaviar, ClinVar, and gnomAD, as well as published articles for supplementary annotation.

### 2.3 In-silico method

An in-silico approach was utilized to establish the structure of the protein in the variant result, with molecular docking employed to ascertain the interactions between the mutant and wild-type receptors. The wild-type receptor and EBV envelope were sourced from UniProt (https://www.uniprot.org/), while the mutant receptor was created by substituting the protein sequence with the variant obtained from WES. Both the wild-type and mutant receptors addressed for 3D modeling by using homology modelling and their structures were evaluated using SWISS-MODEL [10]. Further assessment of these structures was carried out using PremPS for an in-depth understanding [11]. To investigate the interaction between receptors, specifically NRP1, MYH9, and EBV, we focused on the interaction via the EBV envelope proteins gH/gL and gB by using ClusPro webserver for molecular docking [12].

To evaluate the homology in our molecular docking, we devised both qualitative and quantitative analysis methods. For the qualitative approach, we employed the superimposition method to overlay the wild-type and mutant docking models. Quantitatively, we examined the similarity of binding sites. Drawing from a prior study, models are typically considered homologous if they exhibit 75 % similarity in binding sites [13]. However, the protein mutations are acknowledged by structural changes of protein. The cut-off 70 % was addressed for analysis. The quantification of protein homology is determined using the formula:

$$\text{Protein}\;\text{Homology}\;\text{Percentage}\;\left(\%\right)=\frac{a}{b}x100\%$$

Where:

a = Number of matching receptor-ligand interactions between the wild-type and mutant models.

b = Total receptor-ligand interactions observed in the wild-type model.

## 3 Results

### 3.1 Characteristics of subject

This study involves two subjects with young-onset NPC from East Java, Indonesia. Despite the simultaneous onset of the disease in both patients, they presented differing clinical manifestations and rates of progression. The first subject's tumor size was smaller compared to the seconds. Moreover, the second subject exhibited more severe clinical manifestations than the first (Table 1).

**Table 1:** Characteristics of young patient with NPC.

Characteristics	Subject One	Subject Two
Age at diagnosis	14 years 9 months	16 years
Onset of neck mass	±6 months	±6 months
Progressivity of neck mass	Slow	Fast
Epistaxis	Infrequent	Frequent
Tinitus	Infrequent	Frequent
Headache	Infrequent	Frequent
FNAB	Non keratinizing Squamous cell carcinoma, undifferentiated subtype	Non keratinizing Squamous cell carcinoma, undifferentiated subtype
Stage of cancer	III	IVA

Note: Slow: indicating a gradual increase; Fast: indicating a rapid increase. "Infrequent": meaning the symptom occurs rarely; Frequent: meaning it occurs often.

**Fig. 1:**
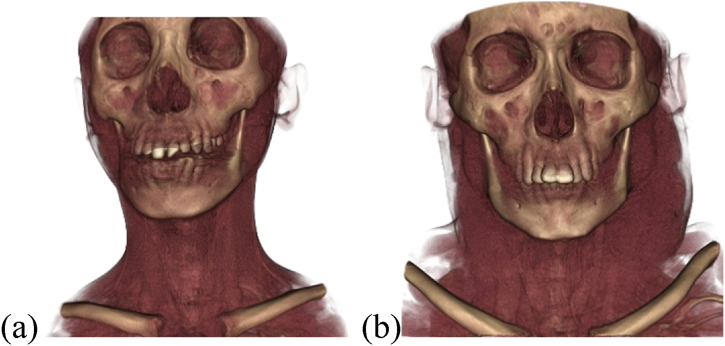
Three-dimensional CT scans. (a) subject 1; (b) subject 2.

Despite having the same disease onset, subjects one and two exhibited differences in the magnitude of NPC mass, as shown by three-dimensional CT scan results. The NPC mass in subject two was larger and metastatic than that in subject one. In subject two, the mass had infiltrated Rosenmüller's fossa, the nasal cavity, auditory tube, parotid gland, and had extended to the cervical region (Fig. 1(a and b), Supplementary Fig. 1 and Supplementary Fig. 2).

Two subjects were referred to the same hospital for further treatment with the same protocol. After one year of follow-up, the first subject underwent serial FNAB, all of which indicated a cancer-free status. However, the second subject passed away three months after their initial visit to our clinic. To gain a better understanding of the differences in clinical manifestations between the subjects, we performed WES.

### 3.2 Genetic variants of receptors identified through WES

We identified four genes associated with nasopharyngeal carcinoma (NPC), including XPC, HCG9, GABBR1, and TP53 (Supplementary Table 2). In whole exome sequencing, 17 % of the genetic variants in subject one are identical to those in subject two. The genetic variants that differ between subject one and subject two account for 6 % of the total variants in subject one (Supplementary Table 3). Further analysis focused on receptors known to interact with EBV, as EBV is a known factor that increases progression and lowers survival rates [4]. These receptors may play a role in the differential manifestations observed in both subjects. Specifically, we analyzed EPHA2, ITGB6, NRP1, and MYH9 (Supplementary Table 1).

We annotated all variants as benign using the annotation tool from the Franklin database. Intriguingly, the variant in subject one not only differs from that in subject two but also results in a protein mutation. Specifically, subject one exhibits variations from subject two in the NRP1 gene (NM_003873.7:c.536T > C, p.Val179Ala), resulting in the alteration of the A allele to the G allele, leading to a substitution of valine with alanine at position 179. Additionally, variations in the MYH9 gene (NM_002473.6:c.4876A > G, p.Ile1626Val) result in the alteration of the T allele to the C allele, leading to a substitution of isoleucine with valine at position 1626 (Supplementary Fig. 4). These genetic variations are common in the general population, particularly among Asians, as evidenced by the Franklin database. Given the disparities in clinical manifestations and disease progression between the two subjects, we conducted further in silico analysis on these variants.

### 3.3 Alterations and instability in protein structure due to NRP1 mutation

To evaluate the three-dimensional structure of the protein, we calculated the protein structure based on template homology. Given that our target protein is already in the database, we employed SWISS-MODEL for our analyses. The NRP1 mutation p.Val179Ala is located in domain, while the p.Ile1626Val is situated in the coiled coil region of MYH9 (Supplementary Fig. 4). For focused structural analysis and subsequent molecular docking, we considered only the locations affected by these mutations. The sequences used were obtained from the UniProt database with several modifications limited to the affected sequences (Supplementary Table 4). It is worth noting that the wild-type protein sequence is available on the UniProt website without any alterations.

This research conducted through investigation into the structural attributes of two models of MYH9. Remarkably, both samples exhibited a consistent level of quality as evidenced by their identical molprobity scores of 0.83 and ramachandran favoured percentages of 98.81 %. Upon close examination of the NRP1 protein models, notable variations were observed. The NRP1 wild-type exhibited a ramachandran favoured percentage of 96.55 %, suggesting a robust structural conformation. On the other hand, the mutant NRP1 p.Val179Ala exhibited a marginal decrease, with a recorded value of 94.83 %. Furthermore, there was a variation in the molprobity scores observed between the NRP1 wild-type (0.91) and the mutant (1.08). The observed divergence between the wild-type and mutant specimens highlights the possibility of structural disparities in the mutant variant (Supplementary Table 5).

In order for strengthening our analysis, we employed PremPS for the purpose of validation. In the present assessment, the mutant NRP1 p.Val179Ala exhibited a ΔΔG value of 2.73, indicating a significant alteration in the protein's stability. Furthermore, it is worth noting that the mutation is located within the core region of the protein. In contrast, the MYH9 p.Ile1626Val protein variant exhibited a ΔΔG value of 0.04, whereby the mutation was observed on the protein's surface conformation (Supplementary Table 6).

### 3.4 NRP1 mutant increase interaction energy with EBV binding, but not in MYH9 mutant

To analyze the interactions between protein mutations and EBV, we utilized molecular docking. However, challenges arose: the binding sites for NRP1 and MYH9 were not found in existing databases or literature, leading us to employ blind docking with ClusPro. We identified 14 models for MYH9-gB in both mutant and wild-type forms and 20 models for MYH9-gH/gL in both mutant and wild-type forms. For NRP1, we identified 20 models across all docking experiments. To further refine our analysis, we labeled the positions as follows: position 0 is NRP1-gB model 0, and position 2 is NRP1-gB model 2. The model with the lowest number model indicates the best binding interaction.

A further complication emerged when comparing the wild-type to the mutant. Because of our dependence on blind docking, solely relying on numerical homology between models was not feasible. To address this, we developed a method that integrates both qualitative and quantitative analyses for each docking model, ensuring distinction between the wild-type and mutant.

**Fig. 2:**
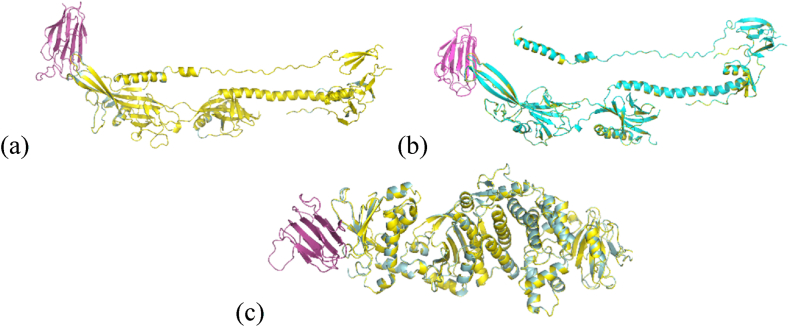
Qualitative Evaluation of Blind Docking Results on both Wild-Type and Mutant Models with the EBV Envelope. a) NRP1-gB 1; b) NRP1-gB 2; c) NRP1-gH/gL 1.

In our initial assessment, we utilized a qualitative evaluation through PyMOL to superimpose all the complexes formed by blind docking. We identified one homolog in NRP1-gH/gL and another in NRP1-gB (Fig. 2(a–c)). For MYH9, there are six models each for MYH9-gB and MYH9-gH/gL (Supplementary Fig. 5). To further refine our findings, we conducted a quantitative evaluation, ensuring that the binding site in the mutants was at least 70 % similar to that of the wild-type. This threshold was based on a previous study that identified molecular unknowns [13]. Based on this criteria, we excluded the NRP1-gB model 2 vs 0 and MYH9-gB 1 vs 1 from our evaluation, as did not meet the required cut-off value (Table 2 and Supplementary Table 5).

**Table 2:** Quatitative Evaluation of NRP1 Blind Docking Results on both Wild-Type and Mutant Models with the EBV Envelope.

NRP1-gB			NRP1-gH/gL		
NO	Model (Wild-Type vs Mutant)	Percentage homolog	NO	Model (Wild-Type vs Mutant)	Percentage homolog
1	0 vs 2	81.81 %	1	0 vs 3	76.47 %
2	1 vs 3	73.07 %			

We subsequently assessed the molecular docking values between the wild-type and mutants for both NRP1 and MYH9. For the model NRP1-gB, the values for 0 vs 2 (number 1) and 1 vs 3 (number 2) were −7 and −20.4, respectively. For NRP1-gH/gL, the value for 0 vs 3 (number 1) was −61.4 (Table 3). In contrast, for MYH9, only minimal differences were observed. For MYH9-gB, the differences were −0.1 and −1.6. Notably, in the case of MYH9-gH/gL, all observed differences equated to zero as indicated in Supplementary Table 6. This highlights the critical role of mutations in the core region in maintaining protein stability and facilitating molecular interactions. Notably, this finding is corroborated by data in Supplementary Fig. 6, which demonstrates a reduced EBV yield in subject one compared to subject two.

**Table 3:** Structural analysis of wild-type and mutant proteins using SWISS-MODEL.

NRP1-gB						NRP1-gH/gL					
Wild-Type			Mutan			Wild-Type			Mutan		
No	Model	Binding affinity	Model	Binding affinity	Δ Binding affinity	No	Model	Binding affinity	Model	Binding affinity	Δ Binding affinity
1	0	−1155.4	2	−1147.4	−7	1	0	−956.0	3	−894.6	−61,4
2	1	−1110.2	3	−1089.8	−20,4						

## 4 Discussion

Young-onset NPC is uncommon, and the presence of the EBV can significantly impact its progression, metastatic potential, and overall prognosis. Globally, EBV infections are pervasive, with a prevalence exceeding 90 % [14]. Notably, there is a rising trend of EBV infections among young individuals in the Asian population [15]. This surge in infections raises concerns since EBV can act as a precursor to NPC. Given these factors, there's an imperative need to devise strategies to prevent the spread of EBV, thereby reducing the potential risk of NPC in younger populations.

In this investigation, we elucidated distinct variants between two young patients with NPC that exhibited differing clinical manifestations, including disease progression and metastatic profiles. This phenotypic disparity may be influenced by the presence of the p.Val179Ala. Our results suggest that this specific allele might play a protective role in the context of NPC. In-depth analysis revealed that this NRP1 variant notably impacts the structural integrity and stability of the NRP1 protein, more so than the p.Ile1626Val. This pronounced effect can be attributed to the location of the mutation: the NRP1 protein mutation is situated within its core, whereas the MYH9 mutation is present on its surface. Historically, mutations localized within the core of proteins are recognized to induce significant alterations to both the structure and stability of the protein, which might further explain our findings [16].

Subsequent to our structural findings, molecular docking was utilized to further characterize the interactions of the mutated proteins NRP1 and MYH9 with EBV. Distinctly, only minor differences were evident between the wild-type MYH9 and its mutant form in relation to the EBV envelope protein gB. In contrast, there were no discernible differences in their interactions with the envelope protein gH/gL. In the context of NRP1, we observed marked differences between the wild-type and the mutant variant. This further confirms our previous discovery regarding the notable structural and stability alterations caused by the NRP1 mutation. The validation provided by EBV yield in RT-qPCR supports the notion that the mutation results in lower EBV levels compared to the normal NRP1.

**Fig. 3:**
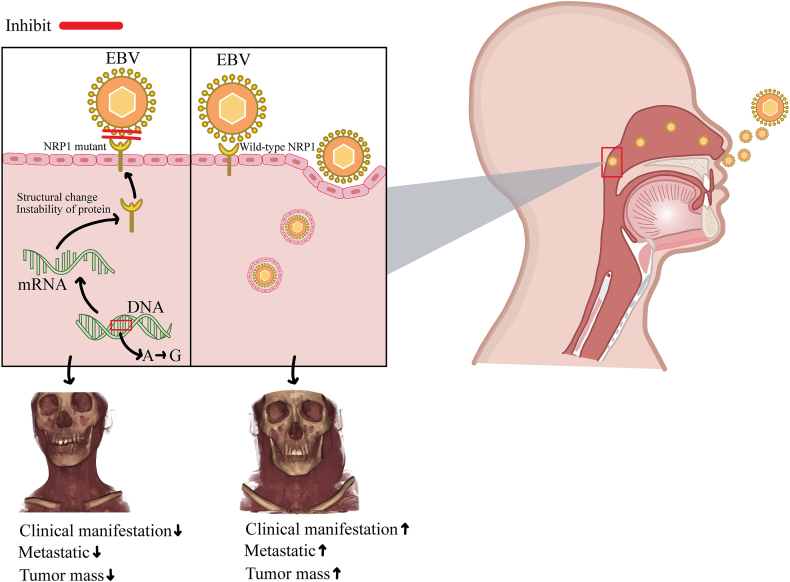
Proposed Mechanism of Interaction between NRP1 (NM_003873.7:c.536T > C, p.Val179Ala) and EBV. The variant NRP1 NM_003873.7:c.536T > C results in the alteration of the A allele to the G allele, leading to a substitution of valine with alanine at position 179 (p.Val179Ala). During protein synthesis, this variant induces alterations in the structure, leading to the instability of the NRP1 protein. This modified structure enhances the energy binding between NRP1 and EBV, which could potentially inhibit the entry of EBV. As a consequence, the presence of this variant might be associated with milder clinical.

The potential impact of a genetic variant on binding energy is discussed concerning its effect on EBV penetration, which could potentially result in an EBV-negative NPC phenotype (Fig. 3). This genetic variant represents an interesting target for further research and interventions. The current observation is consistent with a previous study that showed reduced SARS-CoV-2 entry in the presence of a mutant form of NRP1 [17].

The milder clinical manifestations and reduced metastatic tendencies observed in subject one compared to subject two could potentially be attributed to this particular variant. This aligns with previous findings: survival rates significantly decrease for patients with over 1000 copies/mL of plasma EBV DNA and those testing positive for serum EBV antibodies, as demonstrated by a 3-year survival rate in a prior study [18]. Reinforcing this, research from Indonesia indicates that a higher EBV DNA load obtained from nasopharyngeal brushings is associated with more advanced tumor stages [19]. Furthermore, patients diagnosed as EBV-positive typically exhibit more severe symptoms, faster disease progression, and a higher incidence of metastasis compared to their EBV-negative counterparts [20].

In accordance with the prevailing paradigm, it is widely accepted that mutations occurring in proteins predominantly lead to detrimental consequences. According to previous studies [21], mutations have the potential to either disrupt the normal functioning of a protein or enhance its chances of being broken down. Our research sheds light on the potential of specific protein mutations in offering protection against NPC, which goes against the commonly held belief.

This study presents innovative in silico methods that utilize structural protein measurements and molecular docking techniques to evaluate variant interpretation. Previous studies have predominantly focused on investigating splicing sites, coding regions, non-coding regions, and missense changes [22]. The study presents a significant discovery in relation to the existing guidelines. The observation is made that the current guidelines lack the ability to distinguish between risk and protective variants. The classifications of these variations range from benign to likely benign, uncertain significance, likely pathogenic, or pathogenic [22].

This study underscores the need to reassess, adapt, and develop new protocols for a deeper understanding of genetic variants. However, its findings are limited by a small sample size of only two subjects, necessitating further research with a larger cohort for validation. Enhancements in homology checks for random docking should incorporate both qualitative and quantitative measures, including variables like geometrical similarity or other relevant metrics. Future studies should investigate whether this variant is specific to younger populations, whether its protective role remains consistent in larger populations, and how this mutation affects disease outcomes when accounting for other risk factors.

## 5 Conclusions

A novel method was effectively utilized to shed light on the influence of genetic variations on protein structure and stability, as well as to analyze intermolecular interactions. The variant p.Val179Ala was discovered to induce structural changes, resulting in reduced protein stability. These alterations in conformation were found to increase the binding energy between the protein and the EBV, potentially obstructing EBV entry into cells. This enhanced binding energy could offer a viable explanation for the milder clinical manifestations and reduced metastatic tendencies observed in subject one. Furthermore, these results illuminate the prospects for personalized medical strategies, such as the creation of NRP1 blockers customized to specific genetic profiles. However, additional research is necessary to validate the effects associated with this particular variant, emphasizing the importance of extending this line of inquiry across a broader population.

## CRediT authorship contribution statement

**Sulistyo Emantoko Dwi Putra:** Writing – review & editing, Validation, Supervision, Investigation, Funding acquisition, Formal analysis, Conceptualization. **Farizky Martriano Humardani:** Writing – review & editing, Writing – original draft, Visualization, Validation, Software, Resources, Project administration, Methodology, Investigation, Funding acquisition, Formal analysis, Data curation, Conceptualization. **Hikmawan Wahyu Sulistomo:** Writing – review & editing, Methodology, Investigation, Formal analysis, Data curation, Conceptualization. **Yulanda Antonius:** Writing – review & editing, Methodology, Investigation, Formal analysis. **Jonathan Jonathan:** Writing – review & editing, Validation, Methodology, Investigation, Formal analysis. **Riyan Charlie Milyantono:** Methodology, Investigation. **Artika Uthary:** Writing – review & editing, Methodology, Investigation. **Risma Ikawaty:** Writing – review & editing, Methodology, Investigation.

## Ethical statement

Since the patients were under 18 years old, their parents provided consent for their participation in this study. Additionally, the patients themselves agreed to participate. Written informed consent was obtained from the parents for their children's participation and the publication of their clinical data, photographs, images, and videos. This study was approved by the Ethics Committee of Ibnu Sina General Hospital, Gresik, East Java, Indonesia (Approval No. 02/437.76/2023).

## Data availability statement

Data available in supplementary material.

## Funding

This work was supported by Indonesian Ministry of Research, Technology, and Higher Education for this study (004/SP2H/PT-L/LL7/2023, 018/SP-Lit/LPPM-01/KemendikbudRistek/FTB/V/2023).

## Declaration of competing interest

The authors declare that they have no known competing financial interests or personal relationships that could have appeared to influence the work reported in this paper.
