# Supplementary material for: Genetic variations and clinical significance in young-onset nasopharyngeal cancer: Analysis of EBV interaction with cellular receptor variants and viral glycoproteins
Source: Heliyon. 2024 Dec 13;11(1):e41198. doi: 10.1016/j.heliyon.2024.e41198 (PMC11712000; doi:10.1016/j.heliyon.2024.e41198)
Supplement: Multimedia component 1 [file mmc1.docx]

(a)
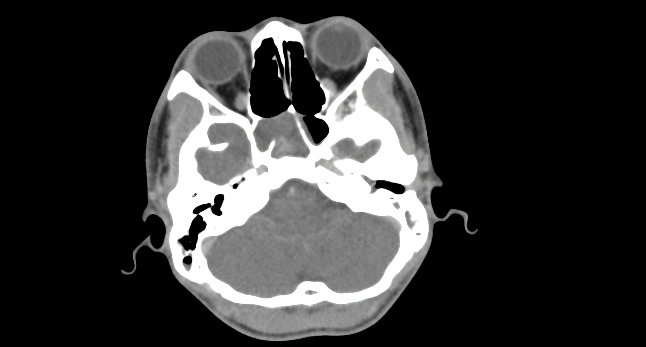
(b)
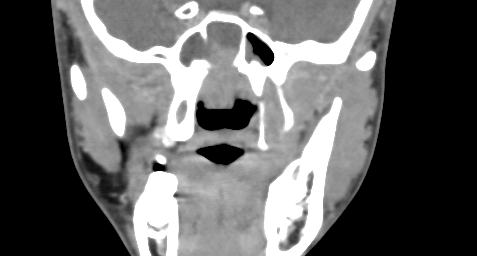


(c)
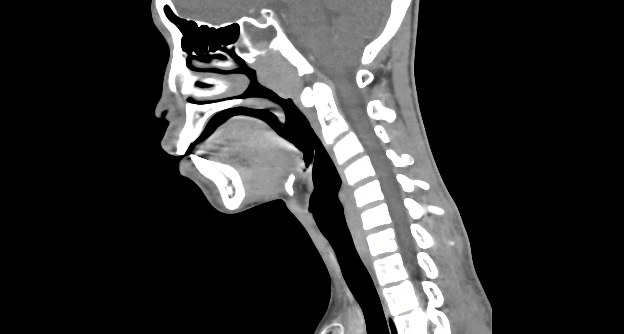
(d)
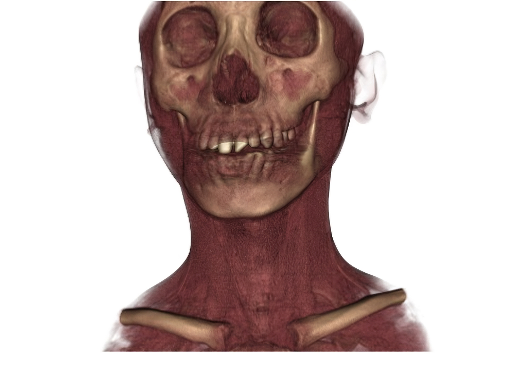


Figure 1. CT scan of young onset NPC subject one. (a) axial plane; (b) coronal plane; (c) sagittal plane; (d) 3D view.

(a)
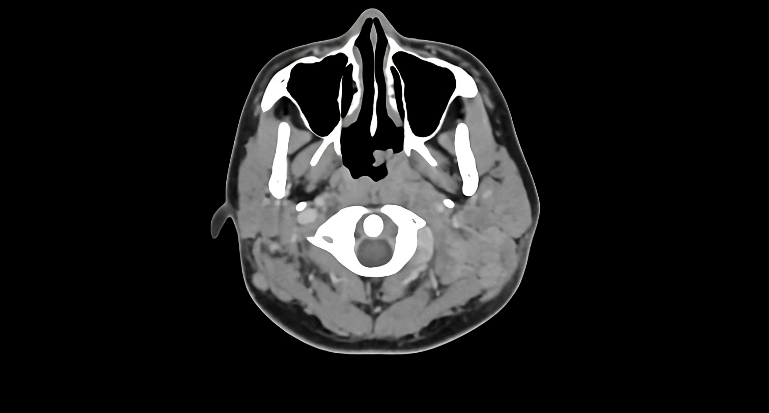
 (b)
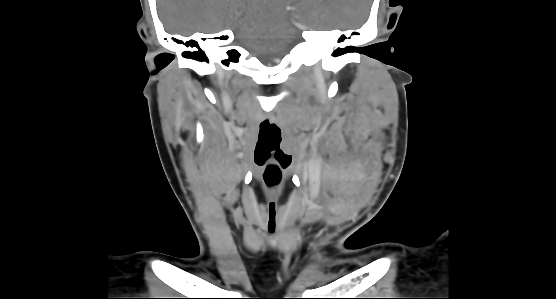


(c)
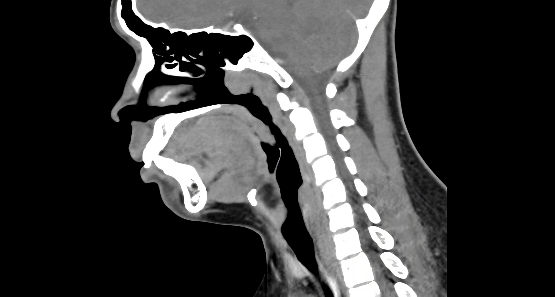
 (d)
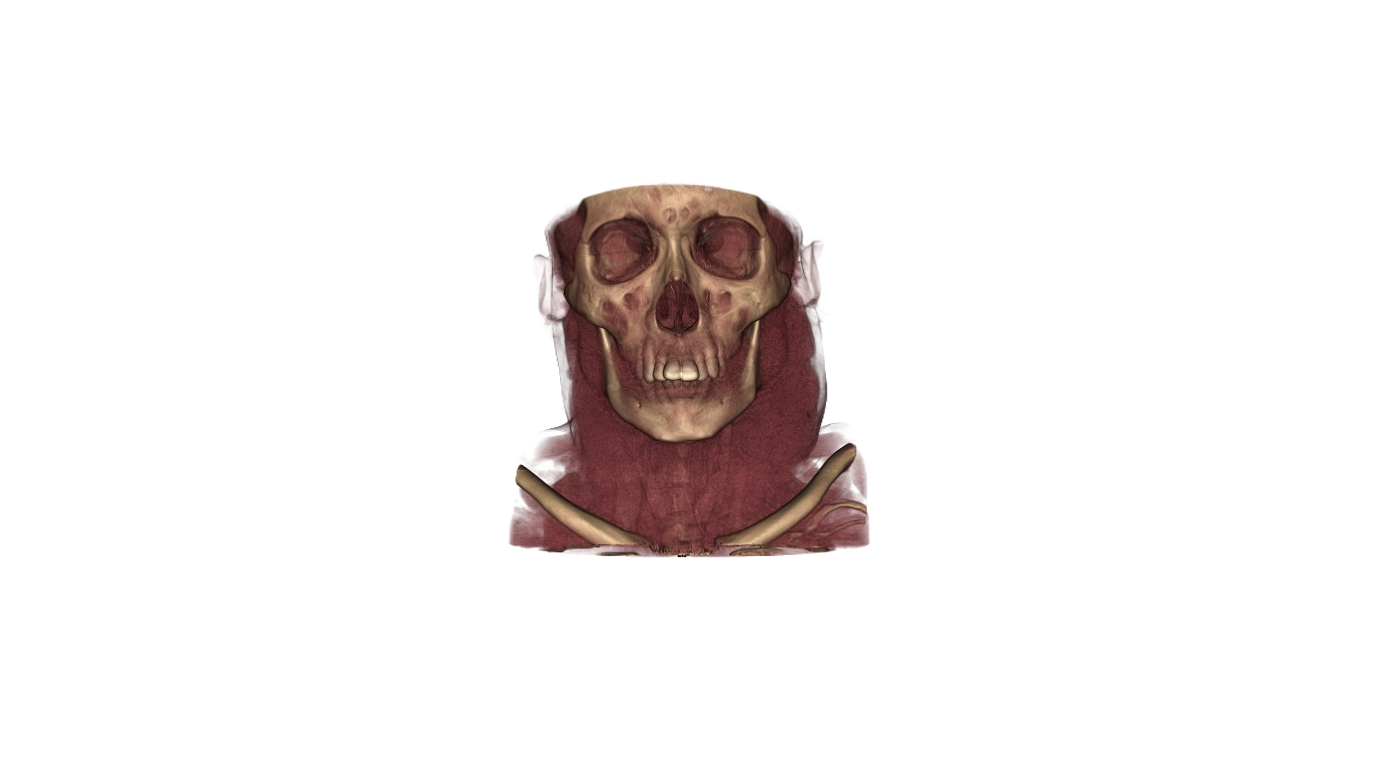


Figure 2. CT scan of young onset NPC subject two. (a) axial plane; (b) coronal plane; (c) sagittal plane; (d) 3D view.

(a)
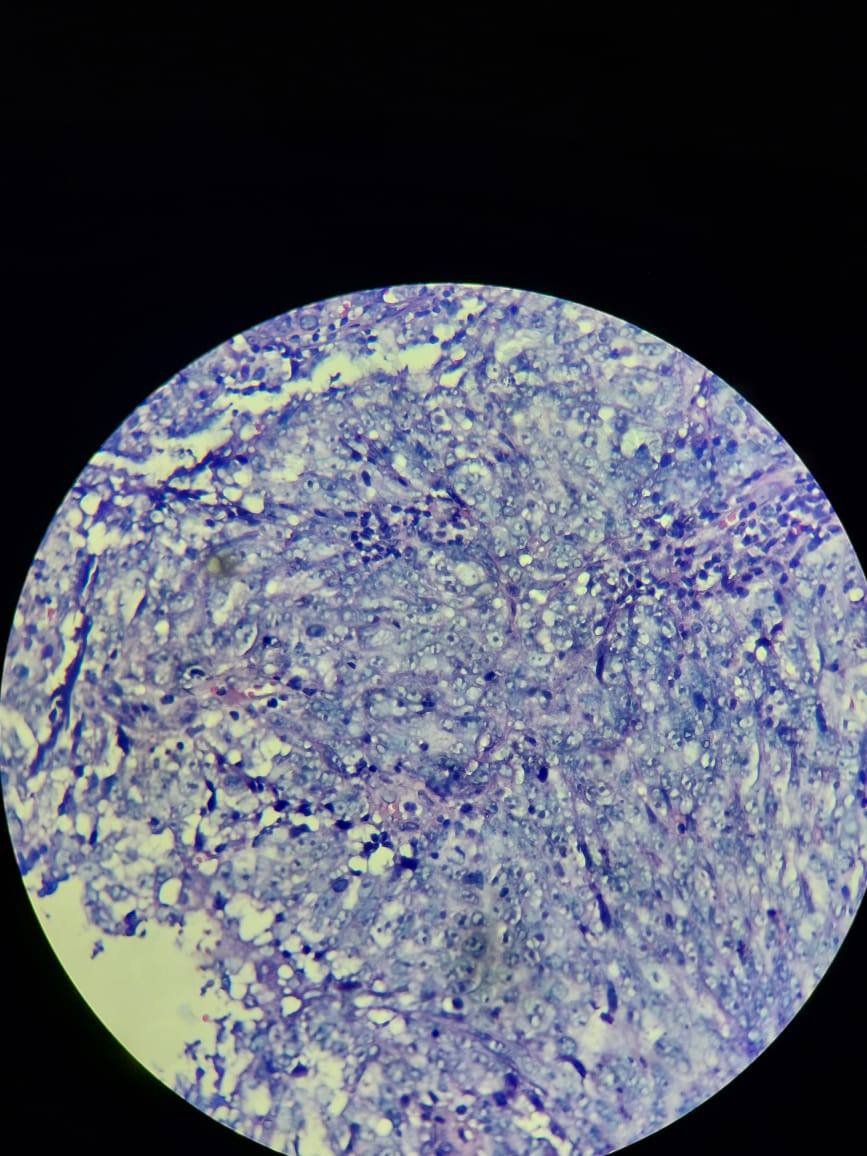
 (b)
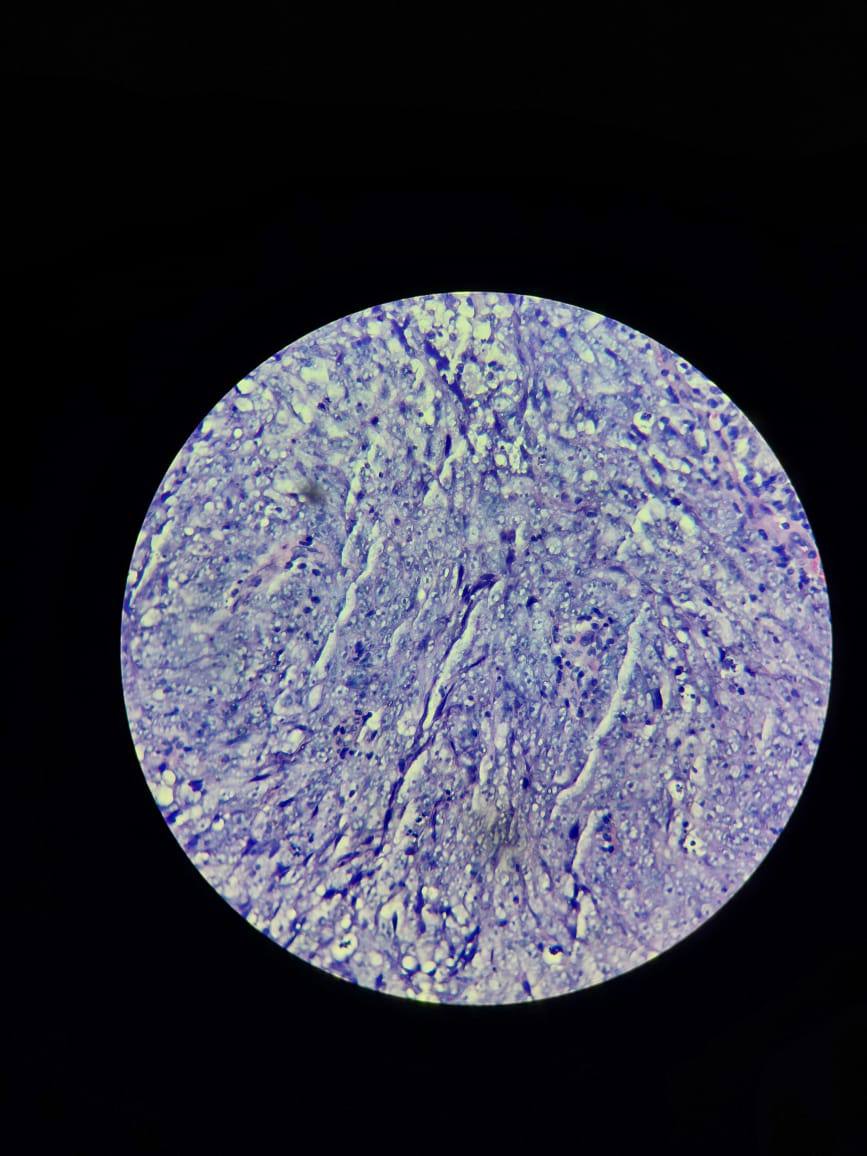


Figure 3. Histopathological examination of NPC in Subject two: (a) Left-side mass magnified 400 times; (b) Right-side mass magnified 400 times.

Owing to hospital regulations, the histopathological examination of Subject One could not be included in this study.

Table 2. Genetic variants contributing to Nasopharyngeal Cancer in both subjects

| Gene | Reference SNPs | Variant | Chromosome | reference |
| --- | --- | --- | --- | --- |
| XPC | rs2228000 | C>T | 3 | (Wu *et al.*, 2017) |
| HCG9 | rs5009448 | T>C | 6 | (Tang *et al.*, 2012) |
| GABBR1 | rs29230 | T>C | 6 | (Tang *et al.*, 2012) |
| TP53 | rs5819162 | CTTT>C | 17 | ClinVar |

Table 3. Total Genetic Variants in Both Subjects

| Chromosome | Subject one | Subject two | Identical Variants Across Subjects | Difference Variants Across Subjects |
| --- | --- | --- | --- | --- |
| 1 | 46.120 (3.250 genes) | 35.064 (3.110 genes) | 8.380 (1.980 genes) | 2.847 (1.072 genes) |
| 2 | 41.242 (2.188 genes) | 29.136 (2.128 genes) | 6.694 (1.402 genes) | 1.907 (750 genes) |
| 3 | 33.540 (1.789 genes) | 24.268 (1.763 genes) | 5.399 (1.142 genes) | 1.624 (631 genes) |
| 4 | 31.938 (1.470 genes) | 22.347 (1.414 genes) | 4.361 (936 genes) | 1.386 (542 genes) |
| 5-6 | 59.558 (3.391 genes) | 42.267 (3.177 genes) | 8.931 (2.005 genes) | 3.179 (1.142 genes) |
| 7-8 | 54.878 (2.878 genes) | 39.804 (2.747 genes) | 8.313 (1.755 genes) | 2.706 (987 genes) |
| 9-10 | 36.022 (2.574 genes) | 49.460 (2.678 genes) | 7.850 (1.708 genes) | 2.622 (954 genes) |
| 11-12 | 54.890 (3.641 genes) | 41.260 (3.464 genes) | 9.950 (2.253 genes) | 3.465 (1.257 genes) |
| 13-15 | 52.393 (2.902 genes) | 29.047 (2.531 genes) | 8.974 (1.861 genes) | 2.530 (979 genes) |
| 16-19 | 54.404 (3.381 genes) | 44.249 (3.949 genes) | 10.308 (2.177 genes) | 3.067 (1.155 genes) |
| 20-22 | 44.635 (3.449 genes) | 24.327 (1.914 genes) | 8.856 (2.098 genes**)** | 3.519 (1.266 genes) |
| 23 (XY) | 10.759 (1.168 genes) | 7.654 (1.060 genes) | 1.711 (525 genes) | 205 (53 genes) |
| Total | 520.379 (32.081 genes) | 388.883 (29.935 genes) | 89.727 (19.842 genes) | 29.057 (10.788 genes) |

Note: In whole exome sequencing, 17% of genetic variants in Subject one are identical to those in Subject two, while 23% of genetic variants in Subject two are identical to those in Subject one. The genetic variants that differ between Subject one and Subject two account for 6% in Subject one and 7% in Subject two.

Table 4. Protein Sequences of NRP1 and MYH9 sourced from UniProt (https://www.uniprot.org/)

| Protein | Location | Sequence protein |
| --- | --- | --- |
| NRP1 | Domain (147-265) | SQNYTTPSGVIKSPGFPEKYPNSLECTYIVFVPKMSEIILEFESFDLEPDSNPPGGMFCRYDRLEIWDGFPDVGPHIGRYCGQKTPGRIRSSSGILSMVFYTDSAIAKEGFSANYSVL |
| MYH9 | Coiled coil (837-1929) | LQVSRQEEEMMAKEEELVKVREKQLAAENRLTEMETLQSQLMAEKLQLQEQLQAETELCAEAEELRARLTAKKQELEEICHDLEARVEEEEERCQHLQAEKKKMQQNIQELEEQLEEEESARQKLQLEKVTTEAKLKKLEEEQIILEDQNCKLAKEKKLLEDRIAEFTTNLTEEEEKSKSLAKLKNKHEAMITDLEERLRREEKQRQELEKTRRKLEGDSTDLSDQIAELQAQIAELKMQLAKKEEELQAALARVEEEAAQKNMALKKIRELESQISELQEDLESERASRNKAEKQKRDLGEELEALKTELEDTLDSTAAQQELRSKREQEVNILKKTLEEEAKTHEAQIQEMRQKHSQAVEELAEQLEQTKRVKANLEKAKQTLENERGELANEVKVLLQGKGDSEHKRKKVEAQLQELQVKFNEGERVRTELADKVTKLQVELDNVTGLLSQSDSKSSKLTKDFSALESQLQDTQELLQEENRQKLSLSTKLKQVEDEKNSFREQLEEEEEAKHNLEKQIATLHAQVADMKKKMEDSVGCLETAEEVKRKLQKDLEGLSQRHEEKVAAYDKLEKTKTRLQQELDDLLVDLDHQRQSACNLEKKQKKFDQLLAEEKTISAKYAEERDRAEAEAREKETKALSLARALEEAMEQKAELERLNKQFRTEMEDLMSSKDDVGKSVHELEKSKRALEQQVEEMKTQLEELEDELQATEDAKLRLEVNLQAMKAQFERDLQGRDEQSEEKKKQLVRQVREMEAELEDERKQRSMAVAARKKLEMDLKDLEAHIDSANKNRDEAIKQLRKLQAQMKDCMRELDDTRASREEILAQAKENEKKLKSMEAEMIQLQEELAAAERAKRQAQQERDELADEIANSSGKGALALEEKRRLEARIAQLEEELEEEQGNTELINDRLKKANLQIDQINTDLNLERSHAQKNENARQQLERQNKELKVKLQEMEGTVKSKYKASITALEAKIAQLEEQLDNETKERQAACKQVRRTEKKLKDVLLQVDDERRNAEQYKDQADKASTRLKQLKRQLEEAEEEAQRANASRRKLQRELEDATETADAMNREVSSLKNKLRRGDLPF |

Red: mutation potition

(a)
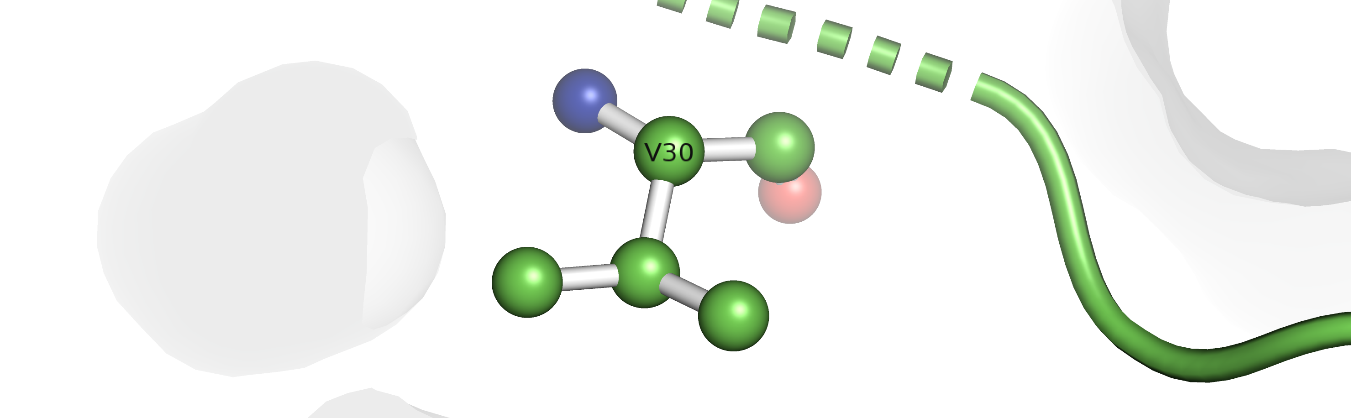
(b)
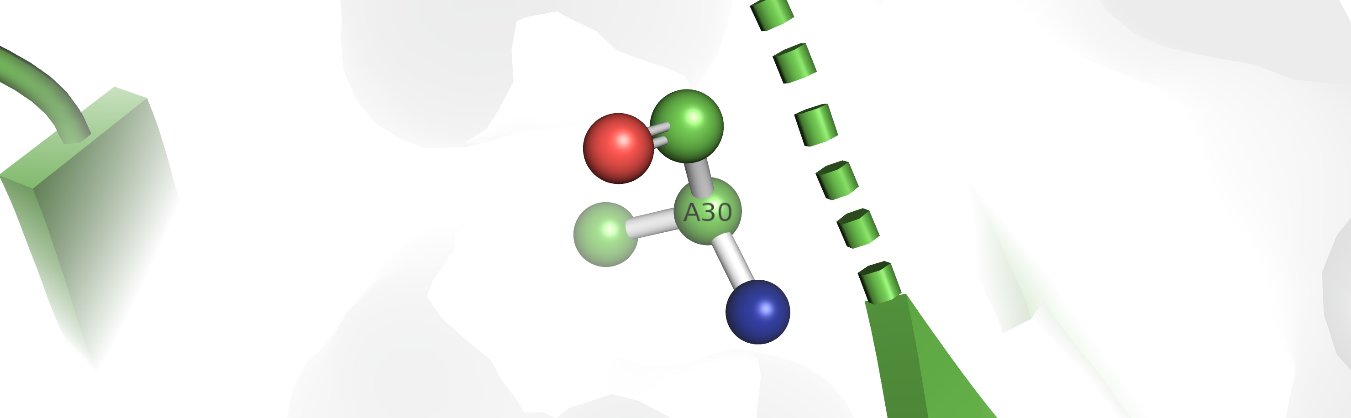


(c)
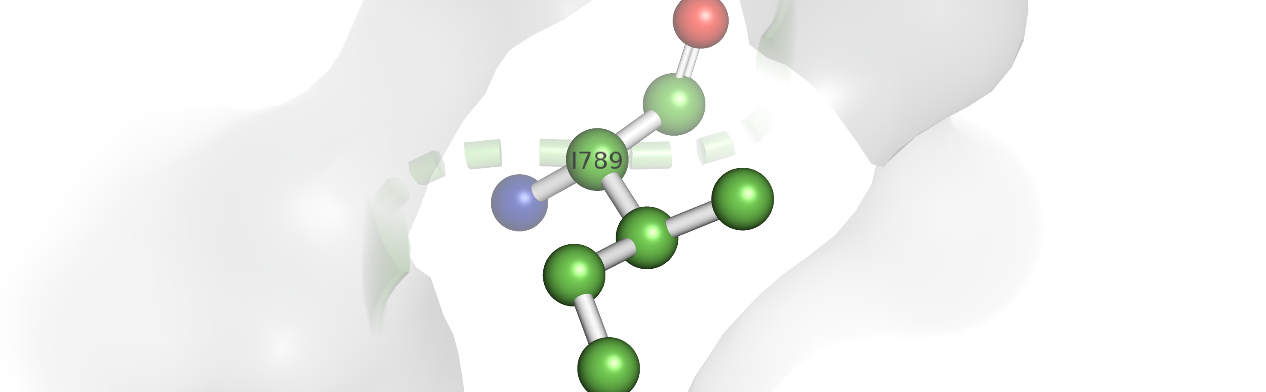
(d)
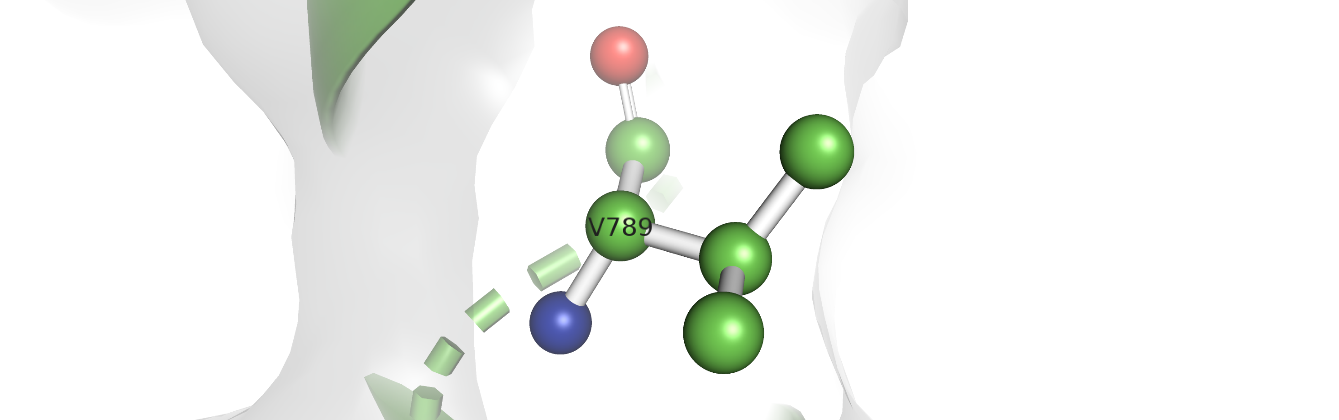


Figure 4. Mutant proteins NRP1 and MYH9. a) NRP1-wild type; b) Mutant NRP1 (p.Val179Ala); c) MYH9-wild type; d) Mutant MYH9 (p.Ile1626Val).

The discrepancy in mutation position numbering arises from the incorporation of structural elements. such as the domain in NRP1 and the coiled coil in MYH9. in the modeling process.

(a)
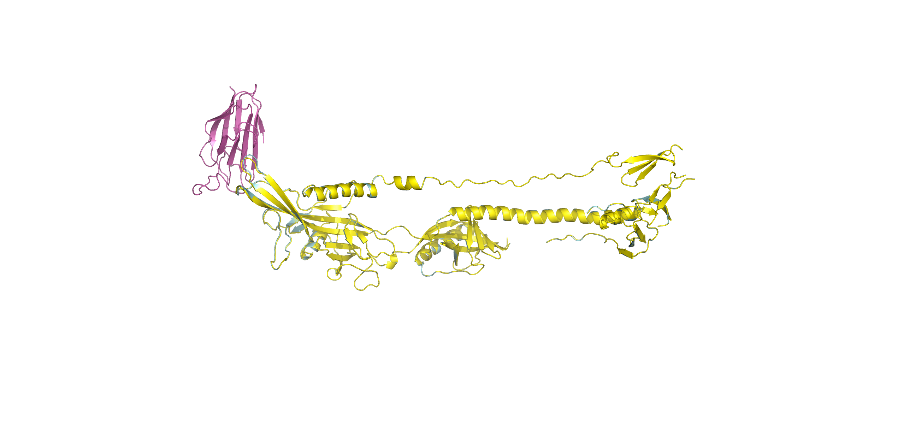
(b)
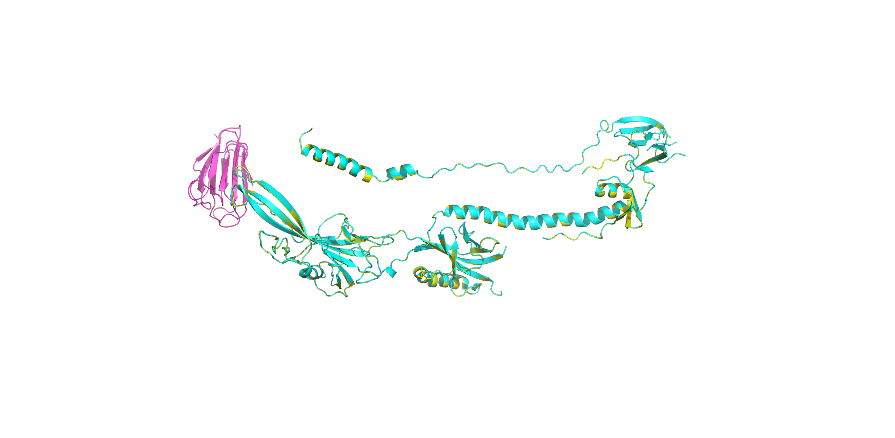


(c)
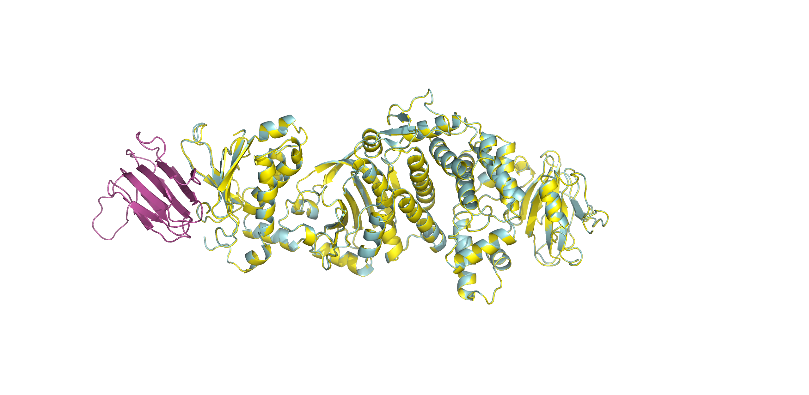
(d)
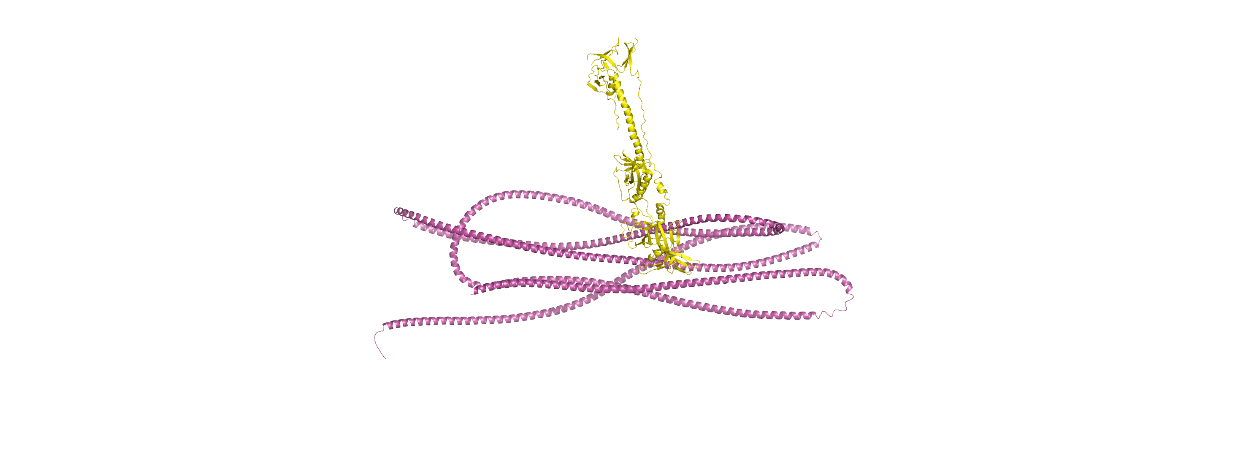


(e)
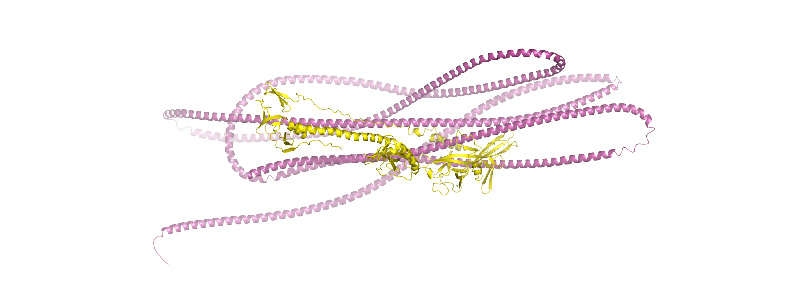
(f)
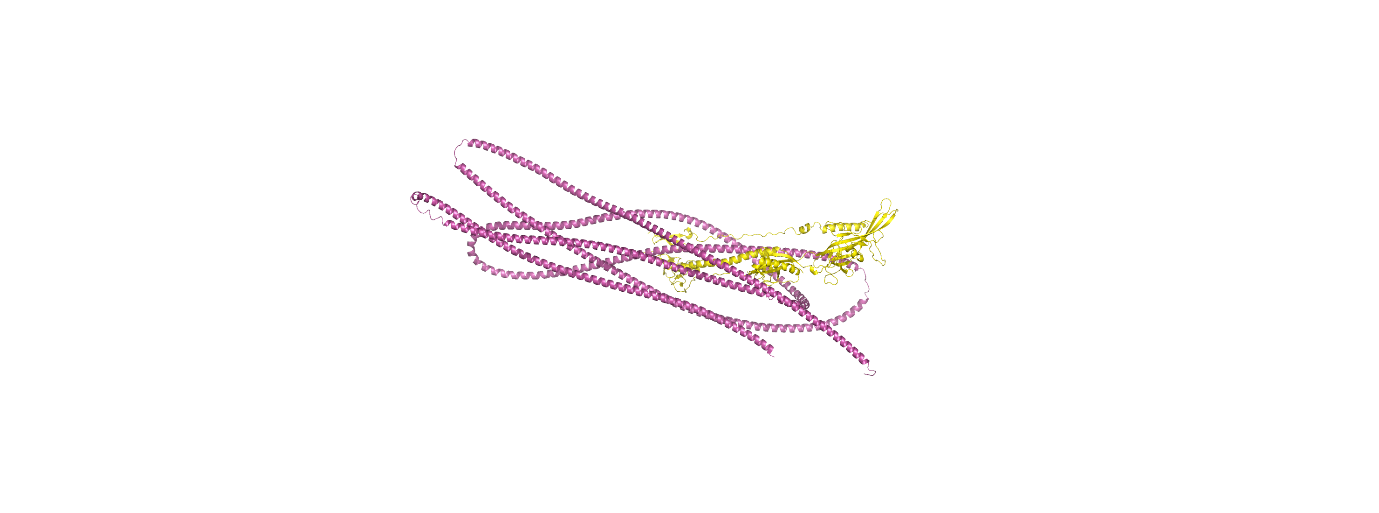


(g)
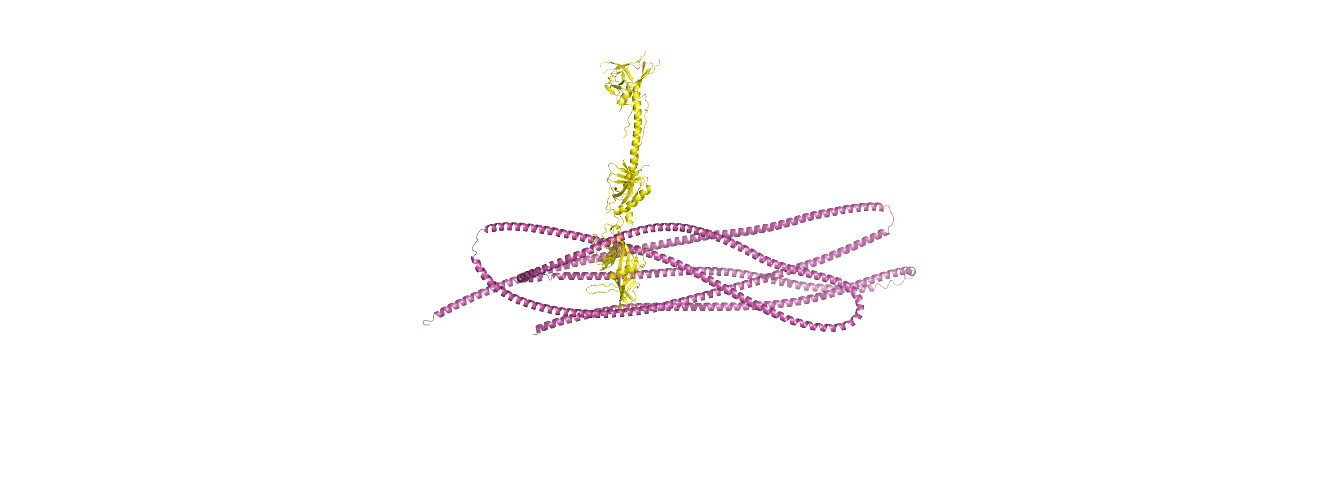
(h)
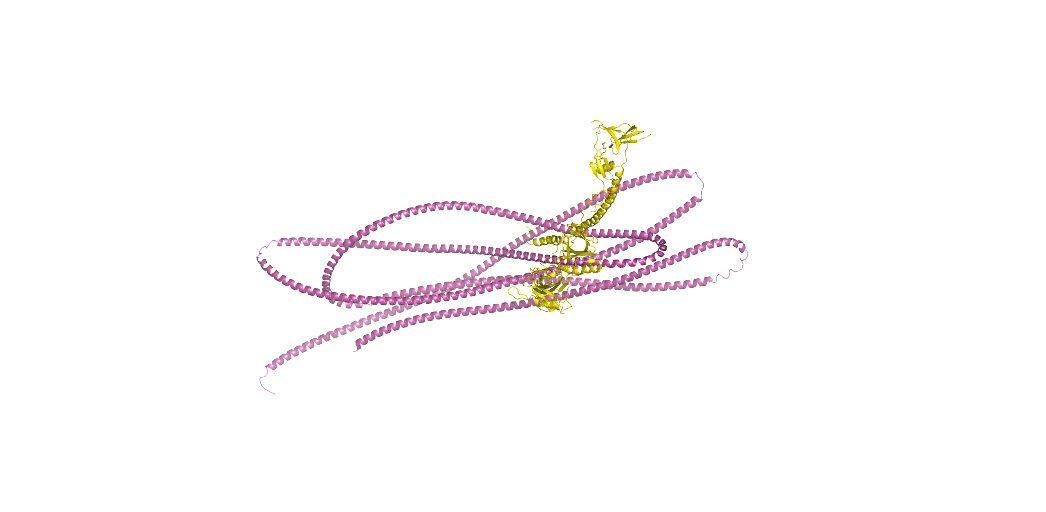


(i)
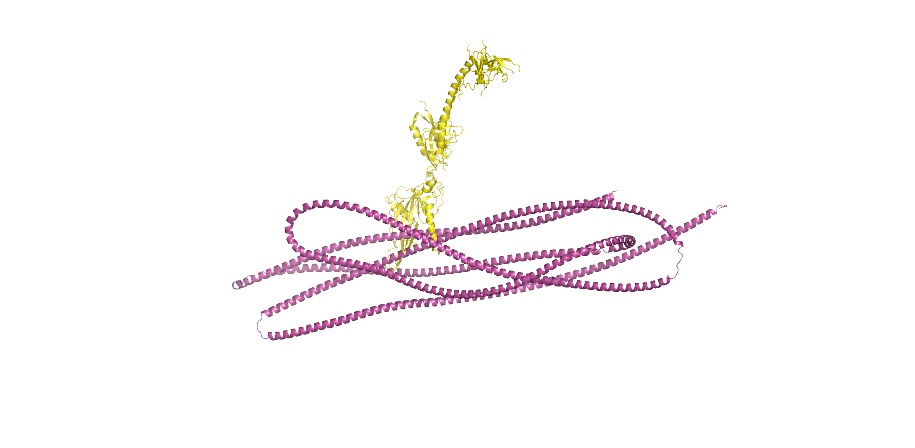
(j)
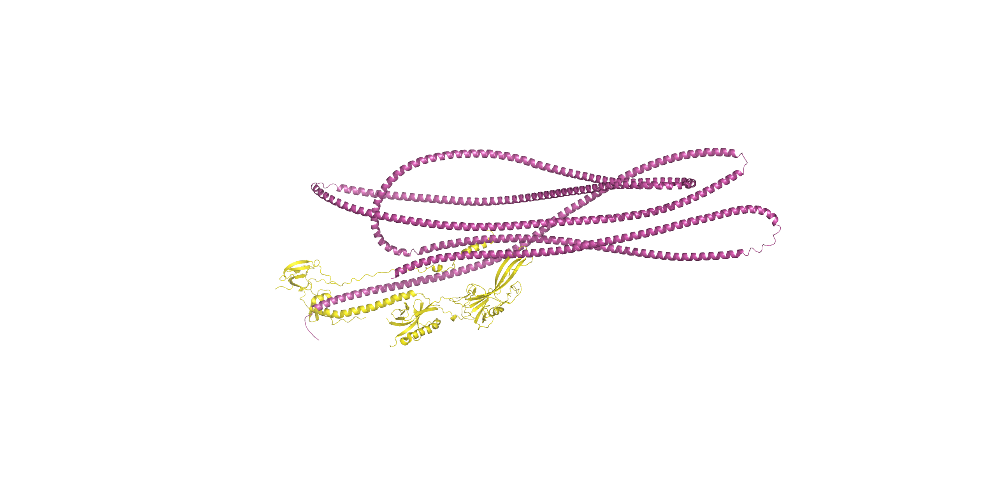


(k)
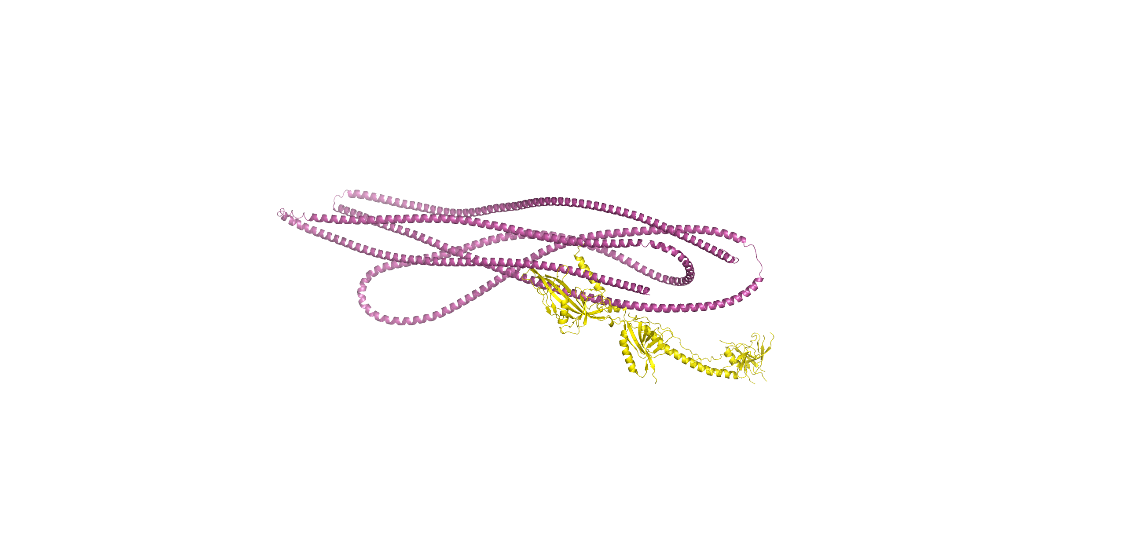
(l)
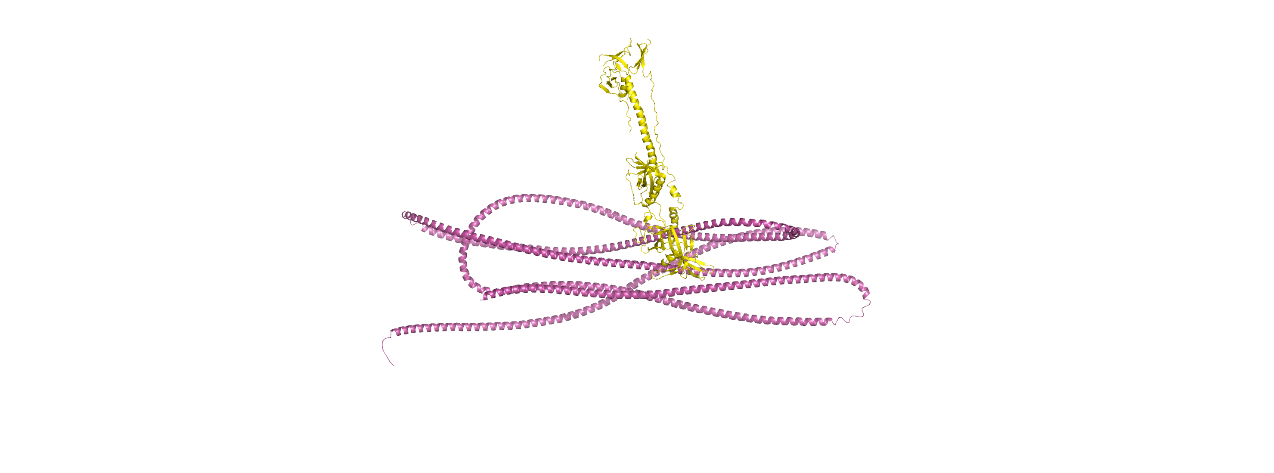


(m)
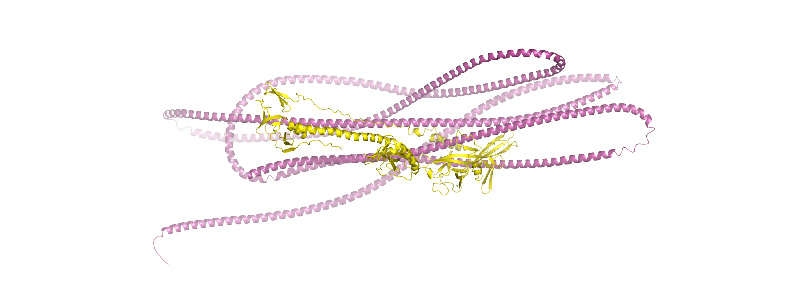
(n)
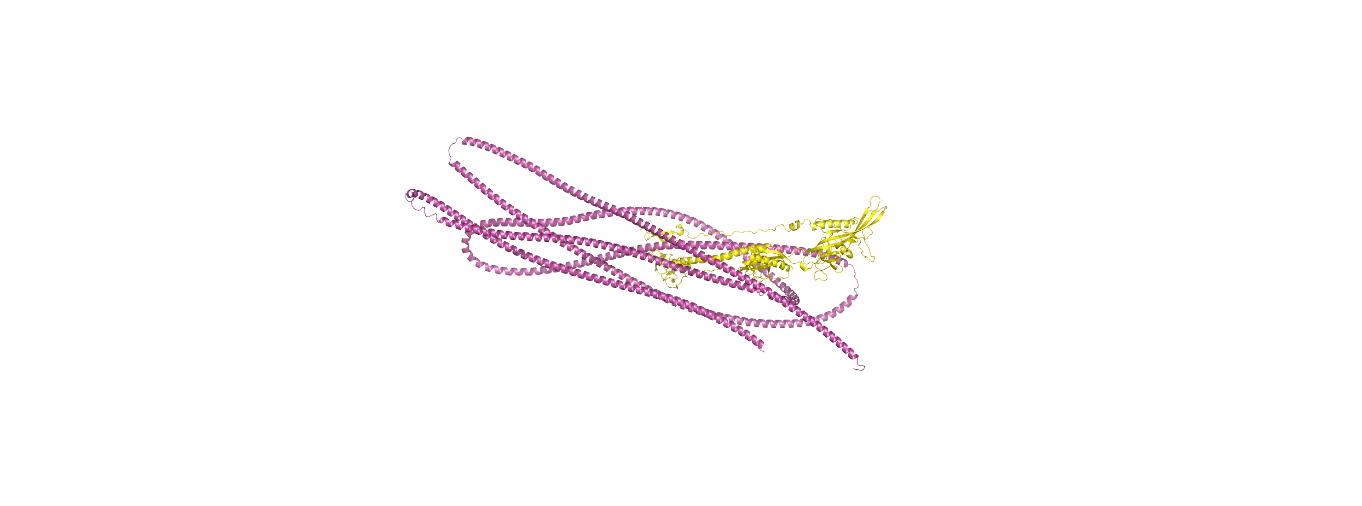


(o)
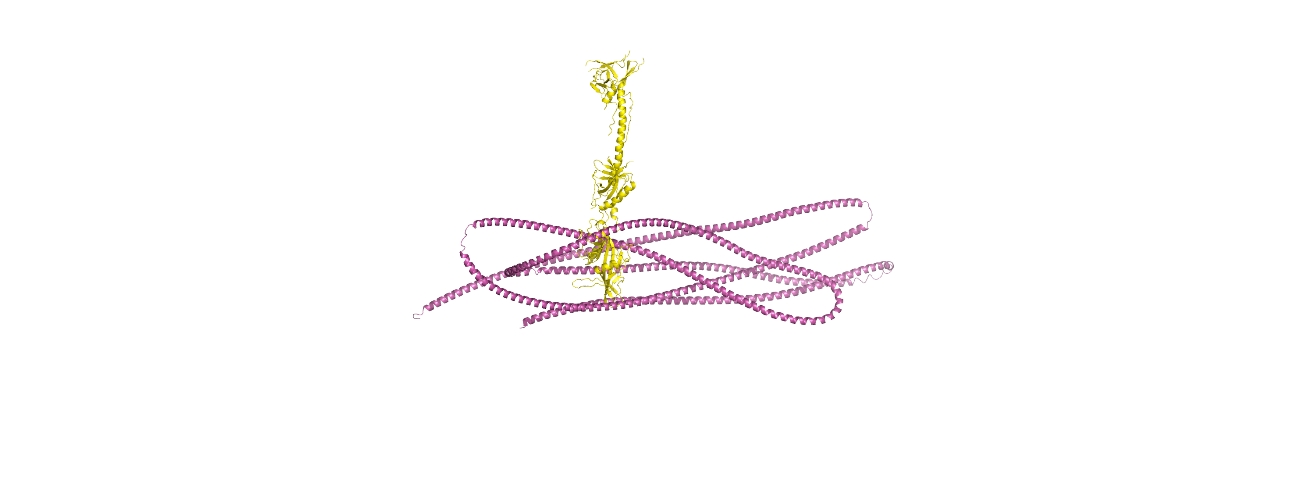
(p)
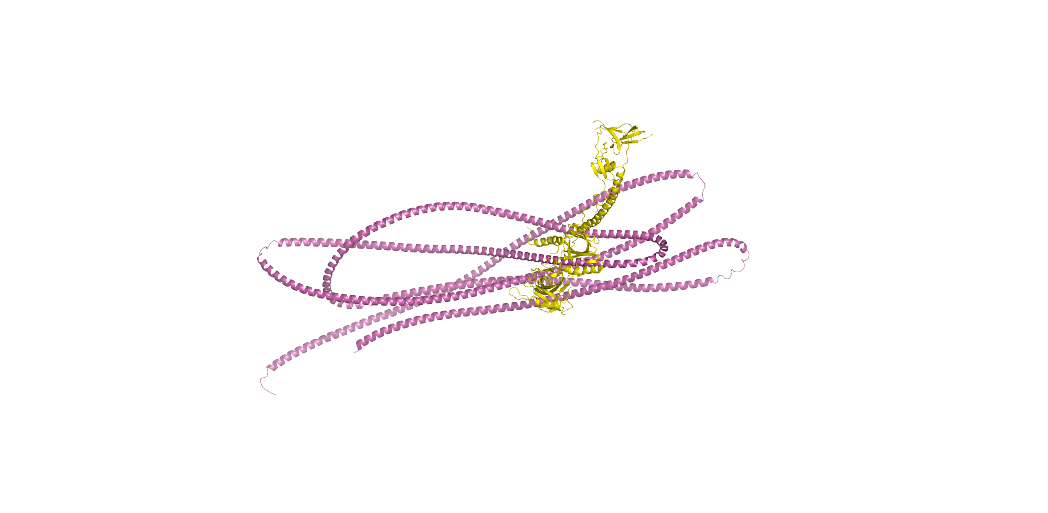


(q)
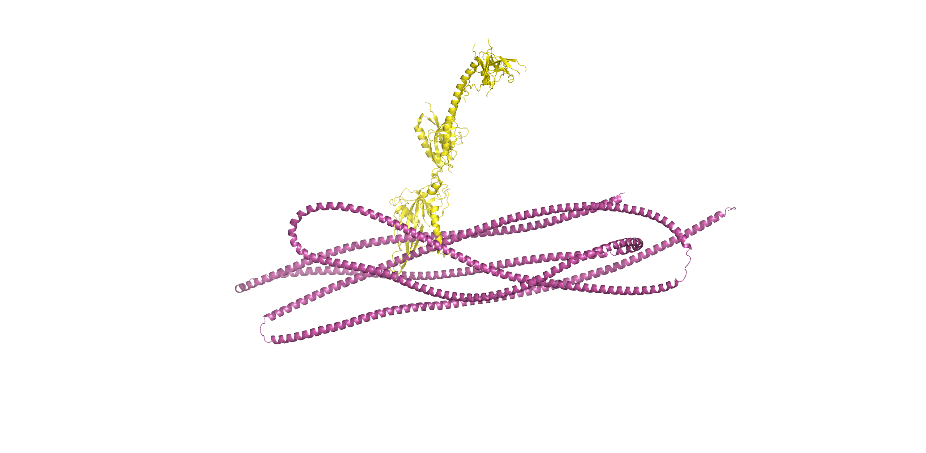
(r)
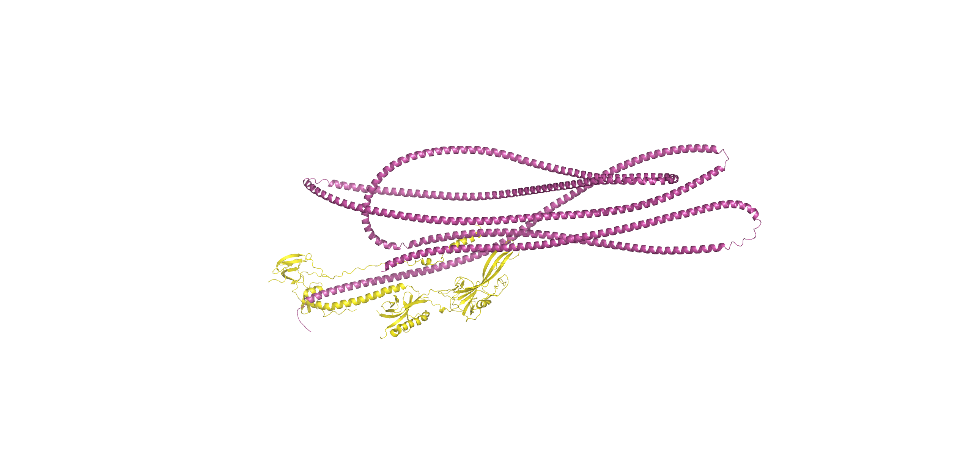


(s)
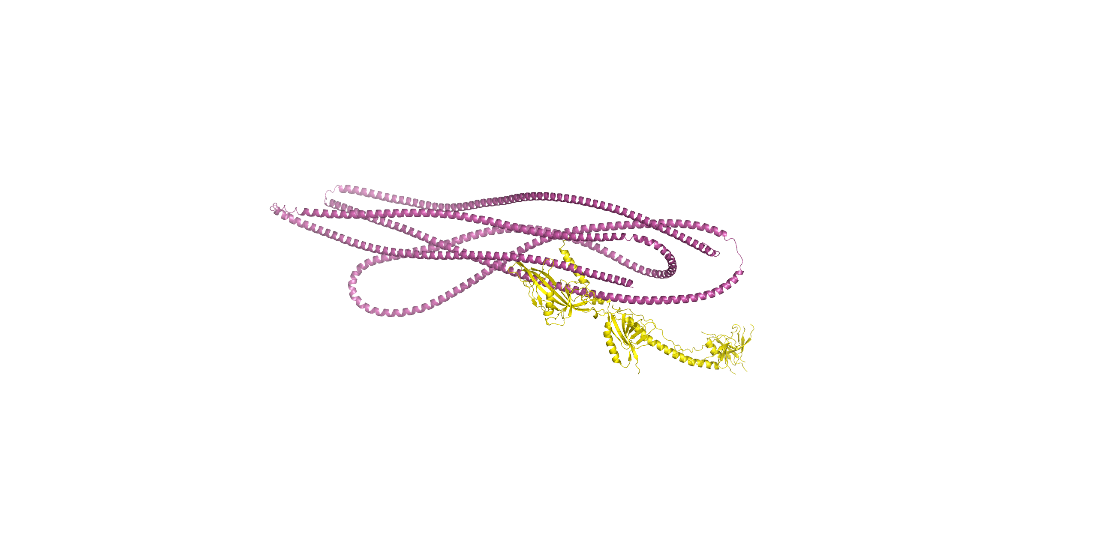


Figure 5. Qualitative Evaluation of Blind Docking Results on both Wild-Type and Mutant Models with the EBV Envelope. a) NRP1-gB 1; b) NRP1-gB 2; c) NRP1-gH/gL 1; d) MYH9-gB 1; e) MYH9-gB 2; f) MYH9-gB 3; g) MYH9-gB 4; h) MYH9-gB 5; i) MYH9-gB 6; j) MYH9-gB 7; k) MYH9-gB 8; l) MYH9-gH/gL 1; m) MYH9-gH/gL 2; n) MYH9-gH/gL 3; o) MYH9-gH/gL 4; p) MYH9-gH/gL 5; q) MYH9-gH/gL 6; r) MYH9-gH/gL 7; s) MYH9-gH/gL 8.

Table 5. Structural Analysis of Wild-Type and Mutant Proteins Using SWISS-MODEL

| Scoring | NRP1 wild-type | NRP1 mutant |
| --- | --- | --- |
| molprobity score | 0.91 | 1.08 |
| ramachandran favoured | 96.55% | 94.83% |
| Scoring | MYH9 wild-type | MYH9 mutant |
| molprobity score | 0.83 | 0.83 |
| ramachandran favoured | 98.81% | 98.81% |

Table 6. Structural Analysis of Protein Mutations Using PremPS

| Receptor | ΔΔG | Location |
| --- | --- | --- |
| NRP1 mutant | 2.73 | Core |
| MYH9 mutant | 0.04 | Surface |

Table 7. Quatitative Evaluation of Blind Docking Results on both Wild-Type and Mutant Models with the EBV Envelope

| NRP1-gB | | | NRP1-gH/gL | | |
| --- | --- | --- | --- | --- | --- |
| NO | Model (Wild-Type vs Mutant) | Percentage homolog | NO | Model (Wild-Type vs Mutant) | Percentage homolog |
| 1 | 0 vs 2 | 81.81% | 1 | 0 vs 3 | 76.47% |
| 2 | 1 vs 3 | 73.07% |  |  | |
| 3 | 2 vs 0 | 65.5% |  |  |  |
| MYH9-gB | | | MYH9- gH/gL | | |
| NO | Model (Wild-Type vs Mutant) | Percentage homolog | NO | Model (Wild-Type vs Mutant) | Percentage homolog |
| 1 | 0 vs 0 | 96.67% | 1 | 0 vs 0 | 97.37% |
| 2 | 1 vs 1 | 51.31% | 2 | 1 vs 1 | 88% |
| 3 | 2 vs 2 | 98.5% | 3 | 2 vs 2 | 82.93% |
| 4 | 3 vs 3 | 95.23% | 4 | 3 vs 3 | 100% |
| 5 | 4 vs 4 | 97.14% | 5 | 4 vs 4 | 97.2% |
| 6 | 5 vs 5 | 98.44% | 6 | 5 vs 5 | 92.68% |

Table 8. Molecular Blind Docking Results for Both Wild-Type and Mutant Models with the EBV Envelope.

| NRP1-gB | | | | | | | NRP1-gH/gL | | | | | | |
| --- | --- | --- | --- | --- | --- | --- | --- | --- | --- | --- | --- | --- | --- |
| Wild-Type | | | | Mutan | | | Wild-Type | | | Mutan | | | |
| NO | Model | Binding affinity | Model | | Binding affinity | Δ Binding affinity | NO | Model | Binding affinity | Model | Binding affinity | Δ Binding affinity |  |
| 1 | 0 | -1155.4 | 2 | | -1147.4 | -7 | 1 | 0 | -956.0 | 3 | -894.6 | -61.4 |  |
| 2 | 1 | -1110.2 | 3 | | -1089.8 | -20.4 |  | | | | | |  |
| 3 | 2 | -1158.3 | 0 | | -1155.8 | -2.2 |  |  |  |  |  |  |  |
| MYH9-gB | | | | | | | MYH9-gH/gL | | | | | | |
| Wild-Type | | | | Mutant | | | Wild-Type | | | Mutant | | | |
| NO | Model | Binding affinity | Model | | Binding affinity | Δ Binding affinity | NO | Model | Binding affinity | Model | Binding affinity | Δ Binding affinity |  |
| 1 | 0 | -1520.8 | 0 | | -1520.8 | 0 | 1 | 0 | -1093.3 | 0 | -1093.3 | 0 |  |
| 2 | 1 | -1836.8 | 1 | | -1851.3 | -14.5 | 2 | 1 | -1000.6 | 1 | -1000.6 | 0 |  |
| 3 | 2 | -1883.6 | 2 | | -1883.5 | -0.1 | 3 | 2 | -1119.6 | 2 | -1119.6 | 0 |  |
| 4 | 3 | -1589.4 | 3 | | -1587.8 | -1.6 | 4 | 3 | -1126.1 | 3 | -1126.1 | 0 |  |
| 5 | 4 | -1489.4 | 4 | | -1489.4 | 0 | 5 | 4 | -1069.8 | 4 | -1069.8 | 0 |  |
| 6 | 5 | -1470.9 | 5 | | -1470.9 | 0 | 6 | 5 | -1040.8 | 5 | -1040.8 | 0 |  |

The RT-qPCR procedure utilized extracted DNA. employing the Bio-Rad T100 thermocycler. This method aims to detect the Bam-Hi-W and BALF5 regions of Epstein-Barr Virus (EBV) circulating in whole blood. Each sample underwent triplet replication to ensure result accuracy. As part of this protocol. researchers also collected blood samples from themselves as healthy controls and used distilled water as a negative control. providing a robust comparison in our analysis.

The thermocycling procedure was set to the following temperature stages: a pre-denaturation stage at 95°C for 5 minutes. followed by denaturation at 95°C for 30 seconds. For the annealing phase. temperatures were set to 53.5°C for the BamHI-W region and 55.2°C for the BALF5 region. each lasting 30 seconds. Extension was carried out at 72°C for 30 seconds. with a final extension at 72°C for 5 minutes. The primer concentration used was 0.5 µM. The primers used were as follows:

Table 9. Sequences of primers used for real-time detection.

| Primer name | Sequence 5’-3’ | reference |
| --- | --- | --- |
| EBV BamHI-W_F | CCCAACACTCCACCACACC | (Miller *et al.*, 2023) |
| EBV BamHI-W_R | TCTTAGGAGCTGTCCGAGGG |  |
| EBV BALF5_F | CGGAAGCCCTCTGGACTTC | (Niesters *et al.*, 2000) |
| EBV BALF5_R | CCCTGTTTATCCGATGGAATG |  |

(a)
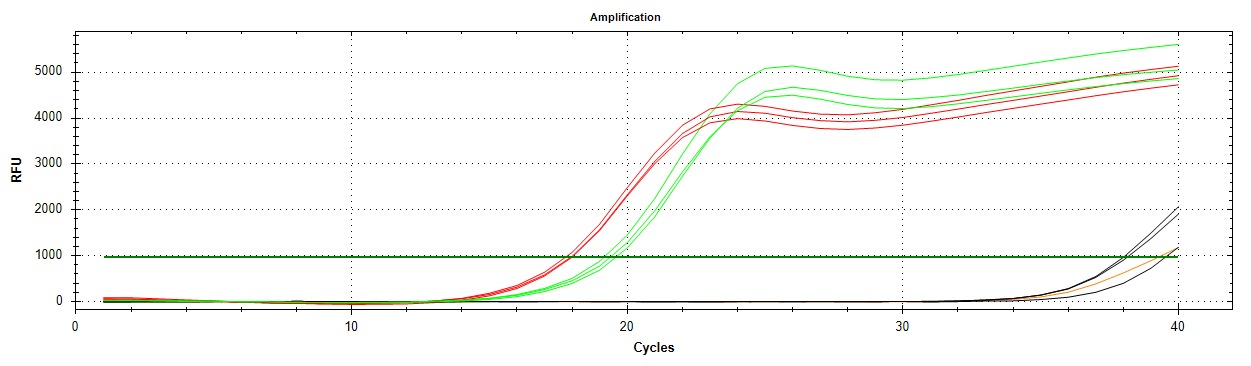


(b)
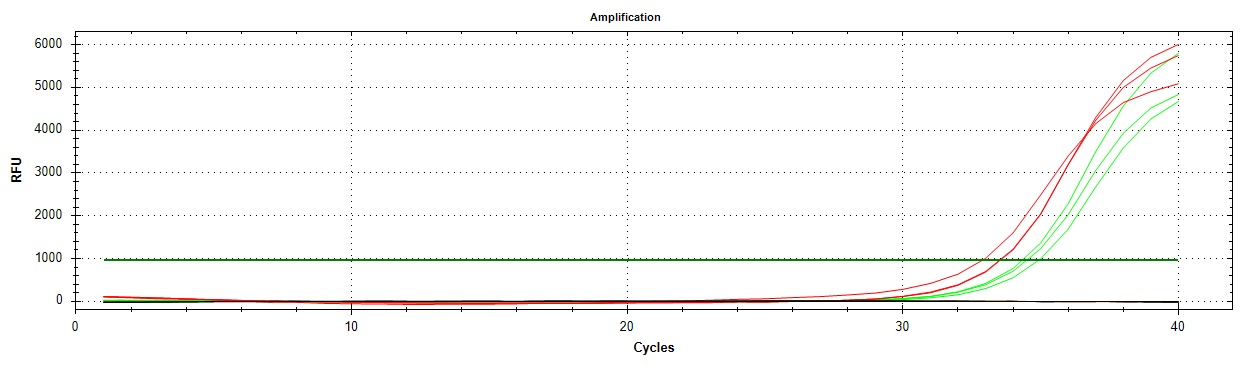


Figure 6. Validation of Epstein-Barr Virus (EBV) in Whole Blood: Subject one and Subject two. This figure presents the validation of EBV in whole blood samples using triplet replication. The data is represented in different colors for clarity: green for Subject one. red for Subject two. black for healthy individuals. and yellow for the negative control. (a) Detection of BamHI-W region of EBV: Cycle Threshold (CT) values for Subject one were 19.57, 19.36, and 19.14; for Subject two, the values were 17.93, 17.98, and 17.74; for healthy individuals, the values were 39.51, 38.12, and 37.99; and for the negative control, the value was 39.23. (b) Detection of BALF5 region of EBV: CT values for Subject one were 34.48, 34.91, and 34.32; for Subject two, the values were 33.53, 33.5, and 32.87; EBV was not detected in healthy individuals or the negative control.

Reference

Bei, J. X. *et al.* (2010) ‘A genome-wide association study of nasopharyngeal carcinoma identifies three new susceptibility loci’, *Nature Genetics*, 42(7), pp. 599–603. doi: 10.1038/ng.601.

Bei, J. X. *et al.* (2016) ‘A GWAS meta-analysis and replication study identifies a novel locus within CLPTM1L/TERT associated with nasopharyngeal carcinoma in individuals of Chinese ancestry’, *Cancer Epidemiology Biomarkers and Prevention*, 25(1), pp. 188–192. doi: 10.1158/1055-9965.EPI-15-0144.

Gao, L. B. *et al.* (2009) ‘Genetic polymorphism of Interleukin-16 and risk of nasopharyngeal carcinoma’, *Clinica Chimica Acta*, 409(1–2), pp. 132–135. doi: 10.1016/j.cca.2009.09.017.

Miller, J. A. *et al.* (2023) ‘Comparison of Real-Time PCR and Digital PCR for Detection of Plasma Epstein-Barr Virus DNA in Nasopharyngeal Carcinoma’, *Journal of Molecular Diagnostics*, 25(7), pp. 490–501. doi: 10.1016/j.jmoldx.2023.03.007.

Niesters, H. G. M. *et al.* (2000) ‘Development of a real-time quantitative assay for detection of Epstein- Barr virus’, *Journal of Clinical Microbiology*, 38(2), pp. 712–715. doi: 10.1128/jcm.38.2.712-715.2000.

Tang, M. *et al.* (2012) ‘The Principal Genetic Determinants for Nasopharyngeal Carcinoma in China Involve the HLA Class I Antigen Recognition Groove’, *PLoS Genetics*, 8(11). doi: 10.1371/journal.pgen.1003103.

Wu, M. Y. *et al.* (2017) ‘Detection of nasopharyngeal carcinoma susceptibility with single nucleotide polymorphism analysis using next-generation sequencing technology’, *Oncotarget*, 8(32), pp. 52708–52723. doi: 10.18632/oncotarget.17085.
